# Ziemssen’s Cyclopædia of Practice

**Published:** 1875-08

**Authors:** 


Cyclopædia of the Practice of Medicine. Edited by Dr. H. Von Ziemssen, Professor of Clinical Medicine in Munich, Bavaria. Vol. iii. Chronic Infectious Diseases. Albert H. Buck, M.D., New York, American editor. New York: William Wood & Co., 27 Great Jones street. 1875. Octavo, cloth, pp. 672. Price, to subscribers, $5.00 per volume.

This great work of Prof. Von Ziemssen has already attained so wide-spread celebrity amongst the American profession, and has been so fully and cordially indorsed by medical critics whose shoe-latchet we are not worthy to unloose, it would be matter of supererogation to attempt here a critical review of its contents. We desire only to bring before such of our readers as are not already subscribers to the work a birds-eye view of its contents, that they may be enabled to know of what they are depriving themselves.

The third volume opens with brief biographical sketches of the authors of the volume, namely: Christian Baumler, Professor of Materia Medica and Director of the Polyclinic of Frieberg, Baden; Arnold Heller, Professor of General Pathology and Pathological Anatomy at the University of Kiel; and Otto Bollinger, Extraordinary Professor of Comparative Pathology at the University of Munich.

The subject of syphilis is first considered by Baumler, and covers 304 pages of the volume. It is elaborately discussed in all of its bearings. Under the head of "prophylaxis" we find the necessity of governmental supervision of prostitutes insisted upon, and the regular examination at stated periods of soldiers and sailors, also recommended. On the subject of marriage: "When a man has enjoyed one, or still better, several years of undisturbed good health after the disappearance of all symptoms of syphilis, he may be allowed to marry; but although under these circumstances there is no direct danger of him infecting his wife, there is no certainty, but only a strong probability, that his offspring will be healthy." Our author believes mercury to possess a positive antisyphilitic power, and recommends its use *immediately* upon the establishment of the syphilitic character of a local lesion. The latter is to be attacked also with fuming nitric acid. During the mercurial treatment the "utmost care" is to be observed to prevent, if possible, salivation. The choice of mercurials is the bichloride, when the state of the digestive organs will admit; otherwise inunction is substituted. Attention has been called elsewhere to the error, on page 290, of *ounces*, which should read *drachms*. The iodide of potassium is not confined to the tertiary stage, but is recommended in the eruptive stage also. The doses recommended are much smaller than many American practitioners deem necessary. It is admitted with regret that the striking results obtained from the iodide are frequently not lasting, and hence it is deemed important that the smallest efficient dose should be used in the begining, so that if long continued the doses required at the last shall not require to be unusually large. The use of liberal quantities of water with the iodide is enjoined, and notice is taken of the suggestion of Sir James Paget, that the efficacy of the remedy is heightened by the conjunction of the carbonate of ammonia. Syphilization is discussed with disapproval.

The second division of the volume, comprised in 239 pages, is Infection by Animal Poisons, by Bollinger, including glanders in the horse, glanders in the human subject, anthrax of animals, anthrax in man, hydrophobia in animals, hydrophobia in man, foot and mouth disease in animals, foot and mouth disease in man, stings of insects, bites of insects and snake bites. In anthrax, thorough extirpation with the knife and subsequent cauterization is enjoined. Quinine and carbolic acid are to be administered internally to the extent of thirty grains of the former and fifteen grains of the latter per diem. "The prognosis of hydrophobia in the human subject is absolutely unfavorable. The malady always terminates fatally. The cases of alleged recovery, which are constantly recurring in the literature of the disease, when subjected to an accurate critical analysis, are invariably found to admit of some other explanation." Prophylaxis: reduction of the number of dogs, by laying the highest possible tax upon them. The curative treatment, as indicated by the prognosis, is absolutely *nil.* The treatment recommended for snake bites is such as is usually employed among us; nothing new suggested.

The third and last division, covering 105 pages, is devoted to migratory parasites, by Heller, comprising echinococcus, cysticercus cellulosa and trichina.

